# Gene content dissimilarity for subclassification of highly similar microbial strains

**DOI:** 10.1186/s12864-016-2991-9

**Published:** 2016-08-17

**Authors:** Qichao Tu, Lu Lin

**Affiliations:** 1 Department of Marine Sciences, Ocean College, Zhejiang University, Zhejiang, 316000 China; 2 Zhejiang Provincial Key Laboratory of Health Risk Factors for Seafood, Zhoushan Municipal Center for Disease Control and Prevention, Zhoushan, 316021 China

**Keywords:** Microbial subclassification, Highly similar strains, Gene content dissimilarity, Genomic fluidity

## Abstract

**Background:**

Identification and classification of highly similar microbial strains is a challenging issue in microbiology, ecology and evolutionary biology. Among various available approaches, gene content analysis is also at the core of microbial taxonomy. However, no threshold has been determined for grouping microorgnisms to different taxonomic levels, and it is still not clear that to what extent genomic fluidity should occur to form a microbial taxonomic group.

**Results:**

By taking advantage of the eggNOG database for orthologous groups, we calculated gene content dissimilarity among different microbial strains based on the orthologous gene profiles and tested the possibility of applying gene content dissimilarity as a quantitative index in classifying microbial taxonomic groups, as well as its potential application in subclassification of highly similar microbial strains. Evaluation of gene content dissimilarity to completed microbial genomes at different taxonomic levels suggested that cutoffs of 0.2 and 0.4 can be respectively used for species and family delineation, and that 0.2 gene content dissimilarity cutoff approximately corresponded to 98 % 16S rRNA gene identity and 94 % ANI for microbial species delineation. Furthermore, application of gene content dissimilarity to highly similar microbial strains suggested it as an effective approach in classifying closely related microorganisms into subgroups.

**Conclusions:**

This approach is especially useful in identifying pathogens from commensals in clinical microbiology. It also provides novel insights into how genomic fluidity is linked with microbial taxonomy.

**Electronic supplementary material:**

The online version of this article (doi:10.1186/s12864-016-2991-9) contains supplementary material, which is available to authorized users.

## Background

Identification and classification of microorganisms is one of the most important but difficult and challenging issues in microbiology, ecology and evolutionary biology. Traditional methods for identification and classification of microorganisms mainly rely on morphological, physiological and biochemical properties of isolated microorganisms [1]. However, characterizing these properties are experimentally very complicated and no quantitative standards can be applied for the obtained descriptive data. Moreover, these properties may differ greatly under different experimental conditions, leading to biased observations of the isolated microorganism. Thus, there have been continuous demands for quantitative approaches to delineate and classify microorganisms by the scientific community, such as methods based on genotypes [2].

For several decades, many efforts have been made to more accurately identify and classify microorganisms, especially at the species level. Among them, DNA-DNA hybridization (DDH) and 16S rRNA gene identity are the two most successful and widely accepted achievements, the former of which is still regarded as the gold standard for microbial species delineation. However, the DDH approach is experimentally tedious and hard to standardize between different laboratories in addition to several other problems, such as that the value obtained with the same pair of strains depends on which is used as probe and which as target [3]. Due to these reasons, 16S rRNA gene analysis has been mainly used in place of DDH for describing new species since the past decades [4, 5]. However, for 16S rRNA gene identity, although it is generally accepted that 97 % or higher sequence identity be used as a cutoff to define microbial species [6, 7], problems have been reported by several labs that 16S rRNA gene identity even cannot distinguish several microbial genera, such as the ones belonging to *Enterobacteriaceae* (particularly *Enterobacter* and *Pantoea*) [4]. And it is now generally accepted that DDH only be carried out when 16S rRNA identity between two strains is 97 % or higher [6, 8].

In the post-genomic era, with more reference genomes getting sequenced by the scientific community, genomic approaches such as in-silico DDH [9], average amino acids identity (AAI) [10], average nucleotide identity (ANI) [11] and multi-locus sequence analysis (MLSA) [12] have been developed. By integrating genomic information, these approaches are proven to be more accurate and reliable in microbial species delineation than 16S rRNA gene identity [13, 14]. Among these approaches, in-silico DDH could be considered as a genomic replacement of wet-lab DDH, for which a 70 % cutoff can be used for species delineation. For AAI and ANI, a cutoff of 94–96 % [10, 11, 15, 16] is generally accepted by microbiologists for their corresponding to 70 % DDH and 97 % 16S rRNA identity, and is becoming a gold genomic standard for microbial species delineation.

Besides the above approaches, gene content analysis proposed as early as in 1999 is another post-genomic analysis at the core of current species definition and has gained success in microbial phylogenetic analysis [17–20]. This approach, although has not gained as wide application as sequence identity based methods, the idea complies several species concepts in microbial systematics such as the recombination theory [21–23] and Cohan’s ecotype concept [24, 25]. These concepts propose that microbial species are formed by acquisition and loss of functional traits through lateral gene transfer and periodic selection, respectively. Such genomic fluidity phenomena plays important roles in microbial genome evolution and identifying closely related organisms such as distinguishing pathogens from commensals [26, 27]. Notably, previous gene content analysis mainly relied on all vs. all pairwise comparison, and reanalysis would always be required when a new genome was added. Most importantly, to our best knowledge, cutoffs for gene content analysis in classifying microorganisms are not yet available.

In this study, we first aim to take advantage of currently available comprehensive ortholog databases such as eggNOG [28], in which orthologous groups are defined by all vs. all clustering approaches, a similar approach as in gene content analysis and thus would simplify the computational procedure for gene content analysis. We then try to address the following two biological questions based on the obtained gene content dissimilarity metrics. First, whether cutoffs could be determined for gene content dissimilarity in classifying microorganisms into different taxonomic groups, ie to what extent genomic fluidity should generally achieve to form a new taxonomic group, eg species? Second, whether gene content dissimilarity could be used for subspecies level classification of highly similar microbial strains? To our best knowledge, current approaches in identifying and subclassifying highly similar microbial strains still mainly rely on phenotypic properties, because approaches based on sequence identity can hardly achieve such purpose due to highly similar conserved genes at subspecies level. As a result, evaluation of gene content dissimilarity using currently sequenced microbial genomes at different taxonomic levels suggested that cutoffs of 0.2 and 0.4 can be respectively used for species and family delineation. Further application of gene content dissimilarity to highly similar microbial strains suggested it as an effective approach in classifying closely related microorganisms into subgroups. This is especially useful in identifying pathogens from commensals in clinical microbiology.

## Results

### Overview of the framework

Although microbial taxonomy at species and higher levels mainly rely on sequence identity approaches such as 16S rRNA gene identity and ANI, identification and classification of highly similar microbial strains still require phenotypic properties of the isolated microorganisms [1]. However, current laboratory approaches may differ greatly from natural conditions and possess limitations of only characterizing a few characteristics of isolated microorganisms. This may lead to misclassification of microorganisms with distinct ecologies, habitats and genotypes. Gene content conceives the notion that the phenotypic properties are ultimately determined by the genes microbial strains harbor. Thus it is expected that the phenotypic differences among different microbial strains can be reflected by gene content dissimilarity.

**Fig. 1:**
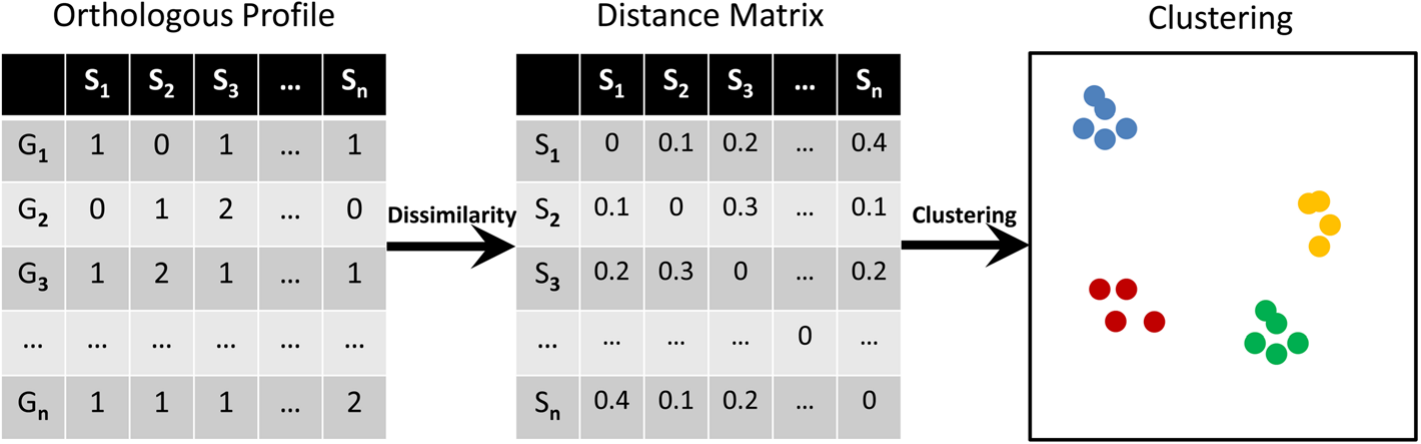
The flowchart of applying gene content dissimilarity for microbial delineation and classification. Three main steps were included. First, orthologous gene profiles were obtained for all selected microbial genomes by searching against the eggNOG database. Second, pairwise gene content dissimilarity as measured by Bray-Curtis dissimilarity was calculated for all pairs of microbial strains. Third, microbial strains were clustered into different groups

Here we proposed a general framework (Fig. 1, also see the methods section for more details) that implemented gene content dissimilarity for potential application in microbial classification, especially highly similar microbial strains that can hardly be distinguished by traditional approaches. Three major steps were included in the framework. First, orthologous gene profiles for microorganisms with complete or near complete genomes were obtained by searching all genes against the eggNOG database. An orthologous gene profile table comprising the abundance of orthologous groups in different microorganisms was generated. Second, weighted Bray-Curtis dissimilarity was calculated as the index representing gene content dissimilarity between different microbial strains. A pairwise distance matrix comprising the gene content dissimilarities among different microbial strains was generated. Bray-Curtis dissimilarity, which was also widely used for microbial functional gene dissimilarities such as in [29], was used here for its weighted property on genetic events such as gene duplication. Third, distance matrix was clustered to group microbial strains into different clusters. It was expected that microbial strains with similar phenotypic properties would be clustered together into the same group for their similar orthologous gene profiles.

### Gene content dissimilarity cutoffs for microbial species and family delineation

**Fig. 2:**
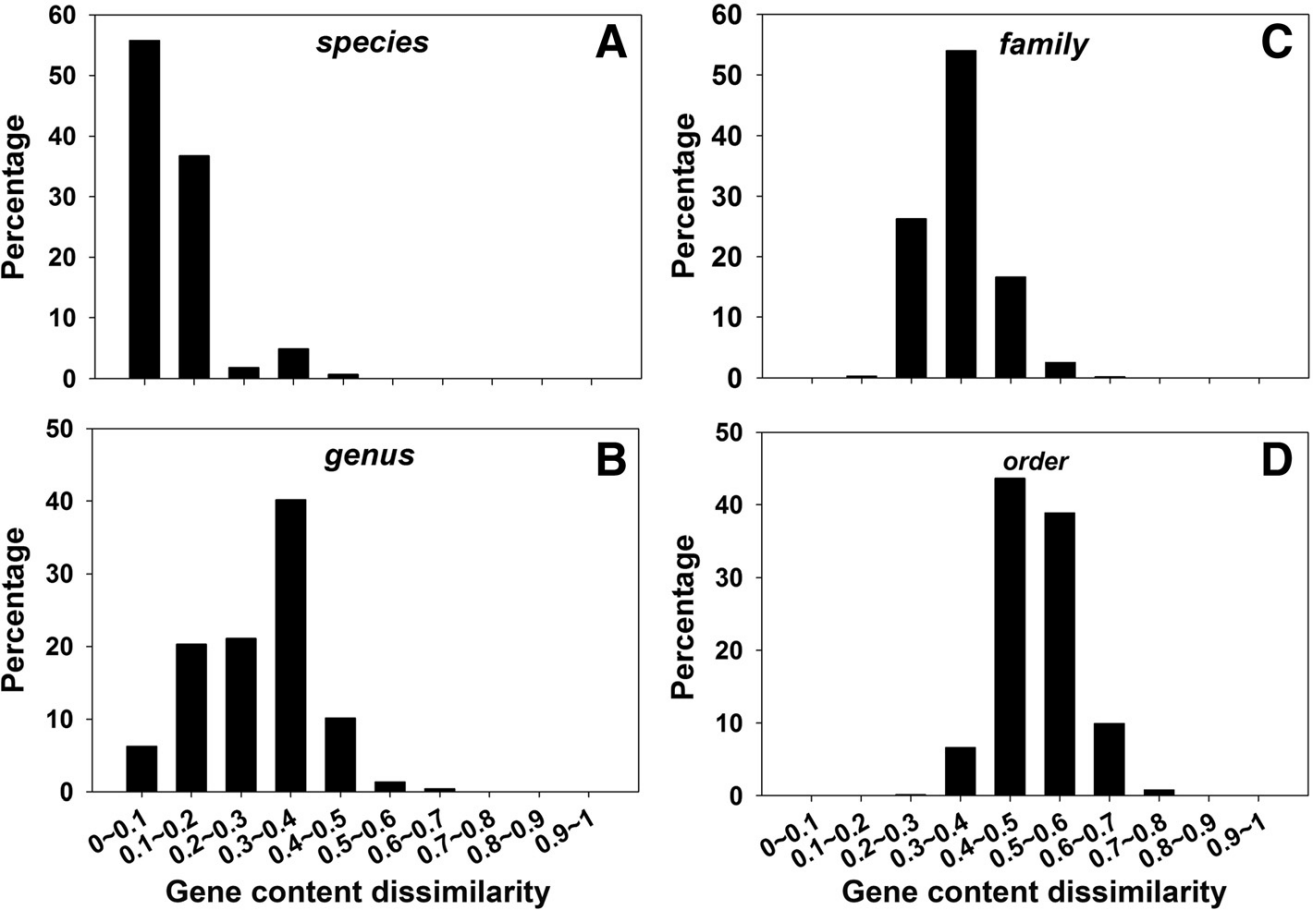
Distribution of gene content dissimilarity for the retrieved microbial genomes at different taxonomic levels, including species (**a**), genus (**b**), family (**c**), and order (**d**). Cutoffs of 0.2 and 0.4 were recommended for microbial species and family delineation, respectively

In order to see whether thresholds can be determined for gene content dissimilarity in classifying microorganisms into taxonomic groups as well as the extent of genomic fluidity in forming microbial taxonomic groups, 2772 complete microbial genomes were recruited and pairwise Bray-Curtis gene content dissimilarities were calculated. Gene content dissimilarity values were then summarized at different taxonomic levels, including species, genus, family, and order (Fig. 2). Interestingly, clear boundaries could be observed for microbial species and family delineation. At the species level, 92.54 % intra-species gene content dissimilarity values fell within 0–0.2 (Fig. 2a). At the genus level, about 26.65 % inter-species gene content dissimilarity values fell within 0–0.2, 61.4 % within 0.2–0.4, and 10.19 % within 0.4–0.5 (Fig. 2b). At the family level, about 80.23 % inter-genus gene content dissimilarity values were within 0.2–0.4 and 16.65 % within 0.4–0.5 (Fig. 2c). At the order level, about 9.69 % inter-family gene content dissimilarity values were smaller than 0.4, and ~90.2 % were within 0.4–0.8 (Fig. 2d). Similar results could still be observed when the most recent eggNOG v4.5 database was used (Additional file 1: Figure S1). Based on these results, it could be found that gene content dissimilarity between microbial strains of the same species were mostly smaller than 0.2, and the value between microbial strains belonging to different families were mostly larger than 0.4. This suggested that gene content dissimilarity cutoffs of 0.2 and 0.4 can be used for microbial species and family delineation, respectively. Notably, similar to 16S rRNA gene identity and ANI cutoffs for microbial species definition, the gene content dissimilarity cutoff was also paradoxical. Strains belonging to the same species were mostly found with smaller than 0.2 gene content dissimilarity. However, not all strains sharing smaller than 0.2 gene content dissimilarity belonged to the same species, because there was still ~25 % possibility that microbial strains with smaller than 0.2 gene content dissimilarity belong to different species but same genus.

### Gene content dissimilarity vs. 16S vs. ANI for microbial species definition

**Fig. 3:**
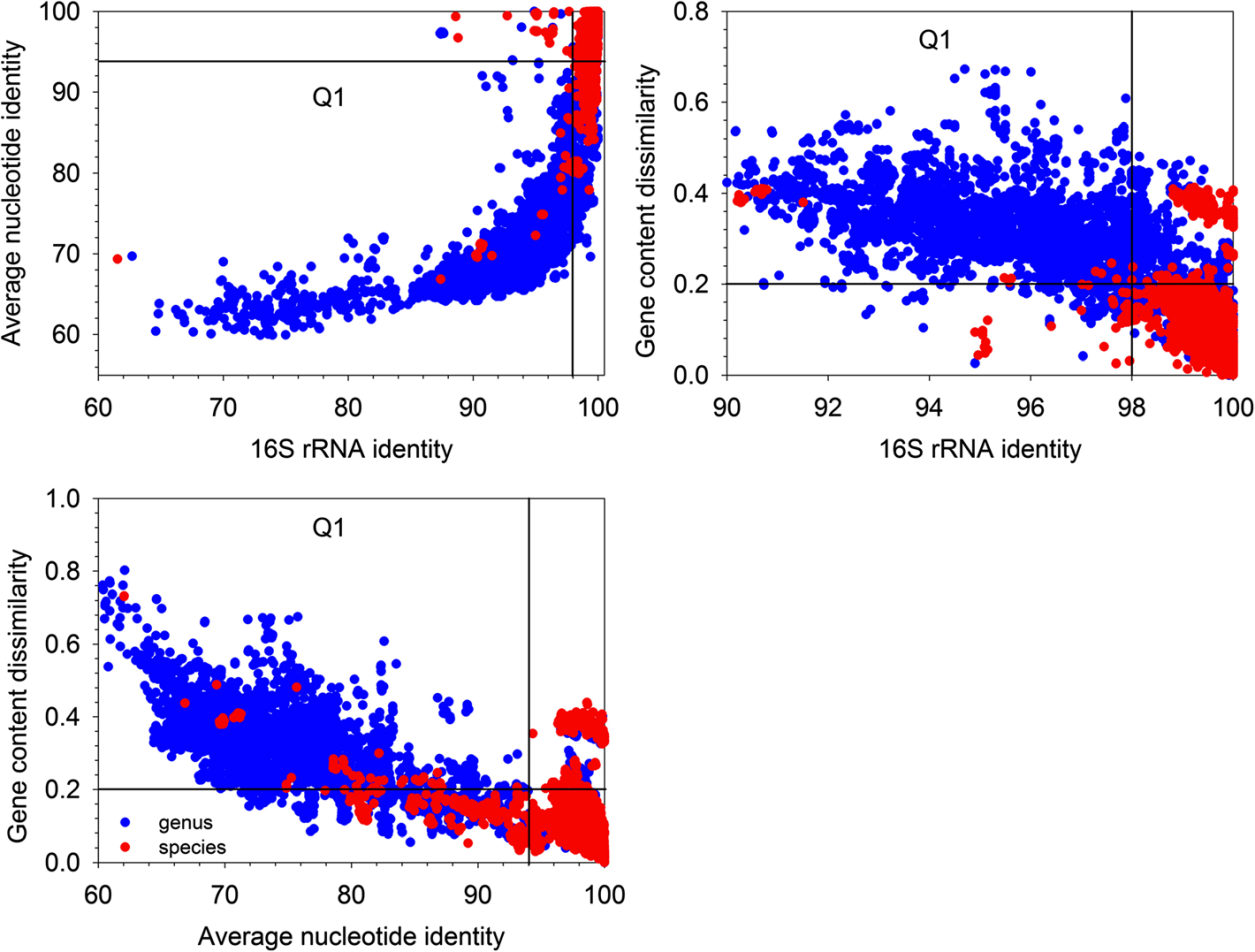
Comparison of 16S rRNA gene identity, ANI, and gene content dissimilarity in microbial species delineation. A cutoff of 0.2 corresponded to 98 % 16S rRNA gene identity and 94 % ANI in species delineation. A total of 5008 intra-species and 8642 intra-genus comparisons were plotted. Red dots falling in the Q1 quadrant were mostly several clostridium strains, for which misclassification may have occurred. Red dots represented intra-species comparisons, and blue dots indicated intra-genus comparisons

One of the most difficult issues in microbial systematics is species identification of newly isolated microorganisms. ANI and 16S rRNA gene identity are two major sequence identity based approaches currently widely used for microbial species identification. ANI cutoff of 94–96 % and 16S identity cutoff of 97–98 % were usually applied for species definition for their corresponding to the gold 70 % DNA-DNA association rate. In order to see whether a similar cutoff can be found for gene content dissimilarity for microbial species identification, intra- and inter-species gene content dissimilarity was compared with corresponding ANI and 16S rRNA gene identity (Fig. 3). Interestingly, gene content dissimilarity cutoff of 0.2 well correlated with 98 % 16S rRNA gene identity and 94 % ANI for microbial species delineation. With 98 % 16S rRNA gene identity, 94 % ANI and 0.2 gene content dissimilarity as cutoffs, about 98.9, 86, and 92.8 % true positive rate was found for microbial strains belonging to the same species, respectively. And about 21.8, 4.7 and 18.8 % false positives were respectively found by misclassifying microbial strains belonging to different species as a same one. A total of 79.4 % microbial strains could be classified to the correct species by all three methods. These results suggested that 94 % ANI was the most conservative method for species definition among all three methods, followed by 0.2 gene content dissimilarity and 98 % 16S rRNA gene identity cutoffs.

To further evaluate the performance of gene content dissimilarity on microbial species delineation, we extracted all microbial strains in the genera with ≥2 species and each species with ≥5 strains. A total of 33 microbial species were evaluated. Significance tests of the orthologous gene profiles of microbial species against other species in the same genus were carried out (Additional file 1: Table S1). The non-parametric multivariate analysis MRPP (multi-response permutation procedure) based on Bray-Curtis dissimilarity distance was performed. As a result, all microbial species subjected to the tests were significantly different from other species in the same genus with *P* ≤ 0.005, except for species *Mycobacterium bovis* (*P* = 0.024) and *Pseudomonas fluorescens* (*P* = 0.018). This suggested that the gene content dissimilarity method proposed in this study can be confidently applied to delineate currently well recognized microbial species.

### Enterobacteriaceae subclassification using gene content dissimilarity

**Fig. 4:**
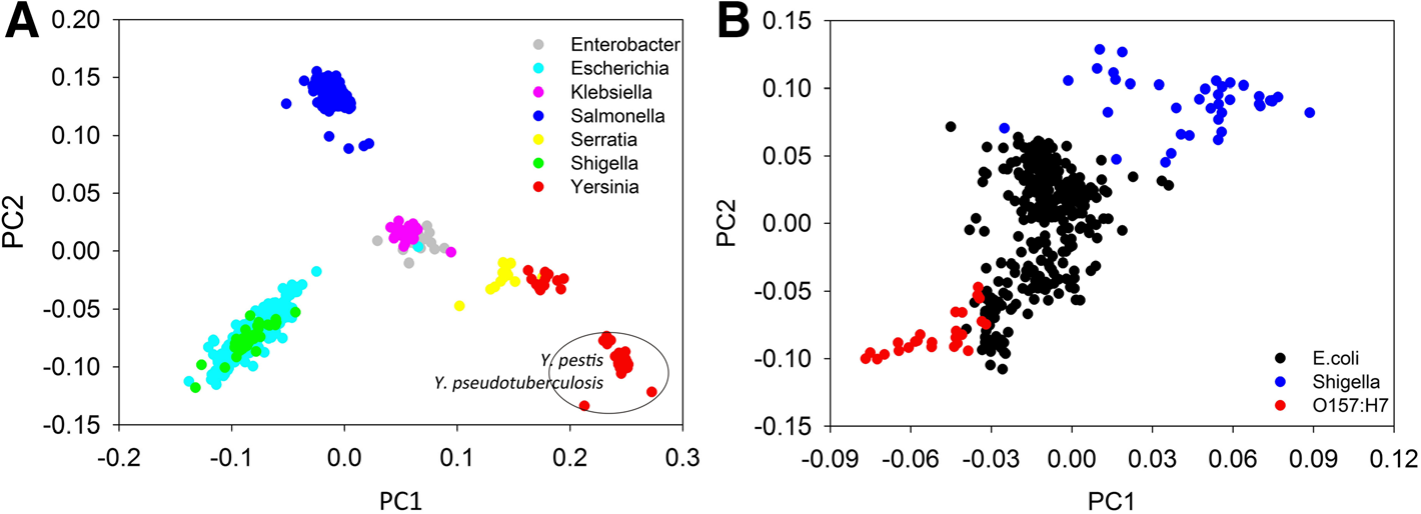
Application of gene content dissimilarity in classifying microbial strains belonging to *Enterobacteriaceae*. **a** PCoA clustering of all selected microbial strains belonging to *Enterobacteriaceae.* **b** PCoA clustering of highly similar microbial strains including *E. coli* and *Shigella*. A clear separation of *Shigella* and *E. coli* O157:H7 from other *E. coli* strains could be observed

In order to see whether gene content dissimilarity can be used as an effective index to classify closely related microorganisms, pairwise gene content dissimilarity was calculated for microorganisms belonging to *Enterobacteriaceae. Enterobacteriaceae* is a relatively well-studied large microbial family with many harmless symbionts as well as a lot famous pathogens. More importantly, representative reference genomes are available for most of them, making it an ideal taxonomic group for testing post-genomic approaches for microbial classification. A total of 916 *Enterobacteriaceae* genomes were recruited, of which 173 were completed genomes and 743 were in draft status. Among these genomes, 14 belonged to *Enterobacter*, 384 to *Escherichia*, 45 to *Klebsiella*, 314 to *Salmonella*, 14 to *Serratia*, 42 to *Shigella*, and 103 to *Yersinia*. As a result, PCoA clustering of gene content dissimilarity showed microbial genomes belonging to the same genus were clustered together and well separated from clusters formed by other genera (Fig. 4a). Notably, microbial genomes belonging to *Enterobacter* and *Klebsiella* were closely clustered, though a trend of separation could be observed. Microbial genomes of two genera, *Escherichia* and *Shigella*, were overlapped and cannot be separated by the first and second axis when other genera were included in the analysis. This was consistent with previous phylogenetic analysis that *Shigella* should be more appropriately classified as a subgenus of *Escherichia*, a phenomenon termed as taxa in disguise [30]. More interestingly, microbial genomes of *Yersinia* were clustered into two distinct clusters, one of which contained *Yersinia pestis*/*Yersinia pseudotuberculosis* genomes, which is consistent with several previous phylogenetic studies based on marker genes including *dnaJ*, *gyrB*, *recA*, *tuf* and *atpD* [31–34]. Genomes in the second cluster were non-*pestis/pseudotuberculosis* genomes and were more closely clustered with *Serratia* genomes.

To further investigate if gene content dissimilarity can also be used to classify closely related microbial strains at subgenus and subspecies level, pairwise gene content dissimilarity was extracted for *E. coli* and *Shigella* strains and then subjected to PCoA clustering (Fig. 4b). This may provide higher resolution in identifying highly similar microbial strains. As a result, *Shigella* strains were well separated from *Escherichia* strains by both first and second axis when other Enterobacteriaceae genera were excluded from analysis. This suggested that although *Shigella* and *E. coli* were highly similar, they were still substantially functionally different and might be considered as different species of *Escherichia*. Interestingly, *Escherichia coli* O157:H7 strains can also be well separated from other *E. coli* strains by the first axis. This indicated that O157:H7 strains harbored markedly different functional capacity from other *E. coli* strains and gene content dissimilarity can be used as an effective post-genomic index to identify O157:H7 strains.

### Streptococcus classification using gene content dissimilarity

**Fig. 5:**
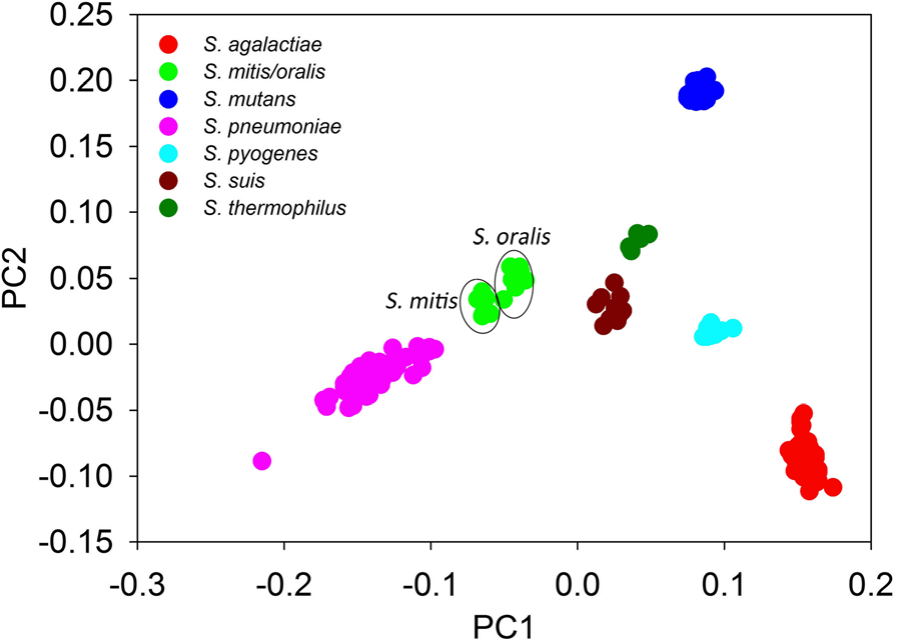
Application of gene content dissimilarity in classifying *Streptococcus* strains. Clear separation of different species into different groups could be observed. Highly similar strains belonging to *S. mitis*, *S. oralis*, and *S. pneumoniae* were also well separated

To further confirm the capability of gene content dissimilarity in classifying highly similar microbial strains, the same approach was carried out to classify microbial strains belonging to the genus *Streptococcus*. Similar to *Escherichia* strains, certain *Streptococcus* species are responsible for many human diseases such as meningitis, pneumonia, septicemia, and sinusitis, while the majority of them are not pathogenic and form commensal microbiota in human body. Although some *Streptococcus* strains can be identified phenotypically and phylogenetically, species such as *Streptococcus pneumonia* can hardly be distinguished from the Mitis group members [35]. Here a total of 84 completed and 199 draft *Streptococcus* genomes were collected and subjected to PCoA clustering based on gene content dissimilarity. Only *Streptococcus* species with more than 5 strains were selected for plotting. These included 83 *S. agalactiae* strains, 7 *S. mitis* strains, 8 *S. oralis* strains, 38 *S. mutans* strains, 119 *S. pneumoniae* strains, 12 *S. pyogenes* strains, 10 *S. suis* strains, and 6 *S. thermophilus* strains. As a result, all these *Streptococcus* species could be well separated from each other by forming individual clusters (Fig. 5). Notably, *S. pneumonia* strains were also well separated from *S. mitis* and *S. oralis* strains. Consistent to previous proposal that *S. oralis* be classified as a member of the *S. mitis* group [36], *S. oralis* and *S. mitis* were closely clustered by gene content dissimilarity based PCoA clustering, indicating that they shared highly similar gene profiles. However, a separation of *S. oralis* and *S. mitis* could still be observed (Fig. 5), suggesting that they might still be two different species or subspecies despite high gene content similarity. This indicated that gene content dissimilarity can also be used as an effective method in distinguishing highly similar *Streptococcus* strains.

### Bacillus cereus subclassification using gene content dissimilarity

We also applied gene content dissimilarity to classify the *Bacillus cereus* group, which is strikingly resistant to any currently available classification systems [37]. A total of 31 compelte genomes and 119 draft genomes belonging to *B. anthracis*, *B. cereus*, and *B. thuringiensis* were recruited. Two analyses were carried out here, including strains with complete genomes and all strains with complete and draft genomes (Additional file 1: Figure S2). Interestingly, clear separation of *B. anthracis* from *B. cereus* and *B. thuringiensis* could be observed in both analyses. The *B. cereus* and *B. thuringiensis* strains with complete genomes could be approximately clustered into three subgroups (Additional file 1: Figure S2A). Such trend of separation could also be observed when more draft genomes were included, but with more vague boundaries (Additional file 1: Figure S2B). This could be due to an increase of subgroups when more draft genomes were added.

## Discussion

Gene content analysis serves as the genomic ground for phenotypic differences and is one of the major post-genomic approaches developed for microbial phylogenetic reconstruction [17–20]. Previous implementation of gene content analysis relies on all vs. all comparison of genes in interested microbial groups, and a reanalysis is needed every time a new strain is added. By taking advantage of recently developed orthologous gene databases such as eggNOG [28], this study suggests that all vs. all comparison for gene content dissimilarity could be approximated by searching against a fixed database. Also, a previous study implementing taxon-specific genes and eggNOG database suggested the usefulness of such strategies in microbial taxonomic classification [38]. Although the approach will suffer potential limitations from not including singleton genes in the database, it is not expected to affect the results because the phenotypic properties expressed by these genes are usually not characterized for microbial classification. In addition, these singleton geneseins are also not subjected to phylogenetic analysis of microorganisms, for which conserved gene families are usually selected [12].

Current sequence identity based approaches mainly focus on the species problem in microbiology, but rarely at other taxonomic levels such as family level. Although gene content analysis has long been applied to reconstruct the phylogenetic relationship of microorganisms, cutoffs have not yet been determined for delineating different taxonomic groups. Recently, Qin et al. applied the concept of percentage of conserved proteins (POCP) to estimate the evolutionary and phenotypic distance between two strains and suggested that a pairwise POCP cutoff of 50 % can be served as a genus boundary for prokaryotic groups [39]. The POPC approach, which relied on all vs. all pairwise identification of orthologous genes, was similar to the one we proposed in this study but more computationally complicated, and seemed not suitable for sublevel classification such as species level. Our results here, interestingly, suggested that gene content dissimilarity could be served as an effective index for microbial subspecies, species and family delineation, but not for genus delineation. Such differences could be due to several reasons, such as the resolution of these two approaches in assigning gene groups, the number of microbial strains recruited in the studies (235 vs. 2772), as well as the possibility that the boundaries between microbial genus and species/family could be relatively vague.

The gene content dissimilarity approach developed in this study complies several species concepts proposed by microbiologists [40], including the recombination theory [3, 40] and Cohan’s ecotype concept [24, 25]. The recombination concept proposes that microbial species are formed by partially exchanging and obtaining homologous and non-homologous genes via lateral gene transfer [21–23]. Recent studies suggest that lateral gene transfer frequently occur to transfer protein-coding genes among microorganisms and is a major evolutionary force for prokaryotes to adapt novel traits such as antibiotic resistance from the environments and other microorganisms in the community [41–44]. The ecotype ecological species concept proposes that prokaryotes form species by adapting to specific environments, for which periodic selection is the major force of cohesion [24, 25]. Genes responsible for adaptive phenotypes are fixed, while less adaptive traits are purged during the periodic selection process. Notably, no matter how different these two concepts are, both theories point out the importance of acquiring and losing of genes/traits in microbial species formation. A question then arise that to what extent microbial species should obtain and/or lose genes/traits to form a new taxonomic group, eg species. Our study addressed this question that a minimum of 0.2 and 0.4 Bray-Curtis gene content dissimilarities should be reached to confidently call species and family, respectively.

More interestingly, application of gene content dissimilarity to highly similar microbial strains/species suggests that gene content dissimilarity can also be served as a powerful index for classifying highly similar microbial strains, although an exact cutoff cannot be determined due to the varied rules in defining microbial subgroups. *Enterobacteriaceae*, *Streptococcus*, and *B. cereus* group are relatively well studied for their wide existence and pathogenic properties of several species. Several species/genera belonging to these groups can hardly be distinguished by traditional approaches, such as *Yersinia* species [45, 46], *E.*coli*/*Shigella [47–49], and *Streptococcus oralis/mitis/pneumonia* [50–54]*.* Among the *Yersinia* species, *Y. enterocolitica, Y. pseudotuberculosis* and *Y. pestis* are pathogenic for mammals. Contrast classification groups are proposed for these three species based on clinical/phenotypic and genotype criteria. Specifically, *Y. enterocolitica, Y. pseudotuberculosis* are classified as one group and *Y. pestis* as another when judging by their clinical and epidemiological features, while DNA-DNA hybridization suggests that *Y. pseudotuberculosis* and *Y. pestis* should be classified as one group or even species for their almost identical chromosomes [45]. Our results supports the later that *Y. pseudotuberculosis* and *Y. pestis* are highly similar at their gene content, consistent with their high DNA-DNA hybridization values. Shigella species are now generally accepted as a clade of the species *E. coli* based on phylogenetic analysis of conserved gene sequences [30, 47, 55]. Due to the failure of phylogenetic identification of Shigella from *E. coli*, they are mainly distinguished by their biochemical and serotype properties. However, recent whole-genome-based approach suggests that all four Shigella species are distinctly different from *E. coli* and form sister species to *E. coli* in the genus *Escherichia* [48]. Interestingly, our results agree with the whole-genome-based study that Shigella and *E. coli* strains are dramatically different from each other, and should be considered as individual species in *Escherichia*. Notably, our results indicated that *E. coli* O157:H7 can even be considered as a different species of *Escherichia* for their divergent gene content from other *E. coli* strains. Similarly, *S. pneumoniae*, *S. mitis* and *S. oralis* are also closely related species and have encountered difficulties in identification using traditional phylogenetic approaches [4], while our results suggested that they could be well separated from each other by gene content dissimilarity. All these results suggest that gene content dissimilarity could be used as an effective index in classifying closely related microbial strains, even at subspecies level.

Notably, although currently available post-genomic approaches are technically different from each other, they are either directly or indirectly linked with the classical DDH method, ie sequence identities. It is therefore not difficult to figure out their high correlation with each other in microbial delineation. What’s interesting here is that these technical differences have addressed different microbial taxonomic problems. For example, the well-known ANI method suggests that microbial species can be defined with an ANI cutoff of 94–96 % [11, 15, 16]. The POCP method, however, suggests a genus boundary for microbial delineation [39]. While the gene content dissimilarity approach we evaluated in this study proposed cutoffs for microbial species and family delineation. No matter how, these approaches have addressed several different questions in microbial systematics and evolutionary biology, such as the relationship between microbial speciation and nucleotide polymorphism, and the extent of genomic fluidity in forming different microbial taxonomic groups. Importantly, current species classification and demarcation are so diverse in metabolic capabilities [56] and ecology [57] that they are neither rooted in evolutionary nor ecological theories [58]. And with the rapid accumulation of genome sequences from so many microorganisms, it is urged by microbiologist that it is now about the time for order in microbial systematics by taking account of both phylogeny and biological signatures [59]. As different indices tried to solve microbial systematics problems in different angles, we herein advocate using multiple indices for confident classification and delineation of microorganisms.

## Conclusions

This study presented a post-genomic approach—gene content dissimilarity, for classification of highly similar microbial strains and as well addressed an interesting evolutionary biology question that to what extent genomic fluidity should occur in forming microbial species and family. Our results suggested that cutoffs of 0.2 and 0.4 gene content dissimilarity could be respectively used for microbial species and family level delineation, the latter of which a general cutoff was not proposed for many years [59] until recently [60]. More importantly, application of gene content dissimilarity showed clear separation of highly similar microbial strains into different subgroups at high resolutions by removing potential noises from other species/genera, ie excluding other species/genera from the analysis. The study provided a genomic mean for identifying closely related microbial strains and could be useful in identifying pathogens from commensals in clinical microbiology, especially when combined with approaches like ANI and 16S rRNA gene identity.

## Methods

### Data acquisition and processing

To evaluate the performance of gene content dissimilarity on microbial taxonomy delineation, a total of 2772 completed microbial genomes were downloaded from NCBI FTP site (ftp://ftp.ncbi.nlm.nih.gov/genomes/archive/old_refseq/Bacteria/). GenBank format genome sequence files and protein sequence files (FASTA format) were retrieved. Full genome sequences and 16S rRNA gene sequences were extracted from GenBank files by PERL scripts implementing BioPerl modules. A full list of the downloaded microbial genomes and accession numbers can be found in Additional file 2.

To test the performance of gene content dissimilarity on distinguishing highly similar microbial strains, both draft and completed genome sequences were recruited for *Enterobacteriaceae*, *Streptococcus,* and the *Bacillus cereus* group, which were three largest microbial groups with known taxonomic problems and many reference genomes available*.* Microbial strains belonging to dominant taxonomic groups were selected for evaluation. A total of 916 *Enterobacteriaceae*, 283 *Streptococcus* genomes, and 150 *Bacillus* genomes were retrieved, respectively. Among the *Enterobacteriaceae* genomes, 14 belonged to *Enterobacter*, 384 to *Escherichia*, 45 to *Klebsiella*, 314 to *Salmonella*, 14 to *Serratia*, 42 to *Shigella*, and 103 to *Yersinia*. The 283 *Streptococcus* genomes included 83 *S. agalactiae* strains, 7 *S. mitis* strains, 8 *S. oralis* strains, 38 *S. mutans* strains, 119 *S. pneumoniae* strains, 12 *S. pyogenes* strains, 10 *S. suis* strains, and 6 *S. thermophilus* strains. Classification and identification problems have been reported for several of these recruited genera/species, such as *Escherichia* vs. *Shigella,* and *S. mitis* vs. *S. oralis* vs. *S. pneumonia.*

### Gene content dissimilarity calculation

We used Bray-Curtis dissimilarity index to measure the gene content dissimilarity among different microbial strains. To do so, we first obtained orthologous gene profiles for each strain by searching all protein sequences against the eggNOG database (v4) [28], which is currently one of the most comprehensive databases for orthologous groups. The eggNOG database was selected also due to its all vs. all clustering procedure in identifying orthologous groups. All COG and NOG orthologous groups were extracted. The program USEARCH (v7.0.1001, usearch_global) [61] was used for database searching for its 10–1250 times faster than BLAST. Coding genes were assigned to these orthologous groups based on the best hit they had with eggNOG database, with an e-value cutoff of 1e-5 and global sequence identity cutoff of 30 %. Microbial strains with less than 1000 genes mapped to the database were excluded from the analysis. A total of 2365 microbial strains were remained for further analysis. Pairwise Bray-Curtis dissimilarity was calculated according to the following function:

$$ B{C}_{ij}=1-\frac{2{C}_{ij}}{S_i+{S}_j} $$

Where *C*_*ij*_ represented the sum of lesser number of genes mapped to each orthologous group, *S*_*i*_ and *S*_*j*_ were the total number of genes mapped to eggNOG database in each genome.

To better illustrate how the Bray-Curtis dissimilarity between two strains was calculated, an example was presented (Table 1). In this example, 9 and 11 genes in strain *I* and *J* were mapped to 4 and 5 orthologous groups, respectively. By taking the lesser number of mapped genes to each orthologous group, the sum of lesser number (ie *C*_*ij*_) of genes mapped to orthologous groups that were found in strain *I* and *J* was 7. Thus a Bray-Curtis dissimilarity of 0.3 could be obtained for strain *I* and *J* according to the above function, ie *BC*_*ij*_ = 1 - 2*7/(9 + 11) = 0.3. The perl scripts for orthologous profile generation and pair-wise Bray-Curtis dissimilarity calculation could be found in Additional files 3 and 4.

**Table 1:** An example showing how Bray-Curtis dissimilarity was calculated between strain *I* and strain *J*. (Note: dissimilarity calculation in real case would be more complex because typical microbial genomes usually comprise thousands of genes)

Orthologous groups	# genes mapped in strain *I*	# genes mapped in strain *J*	*C*_n_^*^	*C*_*ij*_^*#*^	*S*_*i*_^*$*^	*S*_*j*_^*%*^	*BC*_*ij*_&
OG1	1	1	1	7	9	11	0.3
OG2	2	4	2				
OG3	4	2	2				
OG4	0	2	0				
OG5	2	2	2				

^*^ *C*_*n*_ was the number of common genes assigned to the same orthologous group, the lesser number of mapped genes was used

^#^ *C*_*ij*_ was the sum of *C*_*n*_, and represented the total number of genes assigned to common orthologous groups between strain *I* and *J*

^$^ *S*_*i*_ was the total number of genes in strain *I* mapped to orthologous groups in eggNOG database

^%^ *S*_*j*_ was the total number of genes in strain *J* mapped to orthologous groups in eggNOG database

& *BC*_*ij*_ is the Bray-Curtis dissimilarity between strain *I* and *J*

### 16S rRNA gene identity and ANI calculation

Pairwise 16S rRNA gene identity was calculated by the USEARCH (v7.0.1001) program [61]. Global sequence identity was calculated. In the case multiple 16S rRNA gene copies were found in a genome, all of them were subjected to calculation and the average value was used as the identity between two microbial strains.

Pairwise ANI calculation for the downloaded 2772 genomes was carried out by a perl script obtained from https://github.com/chjp/ANI. This script employed the same algorithm and output the same result as the JSpecies program [16]. The program BLAST (v2.2.25) was called in the script for ANI calculation.

### PCoA clustering and significance tests

We employed PCoA clustering methods for better visualization of our results. In fact, many other clustering programs should also work in separating microbial strains into different groups. The non-parametric multivariate analysis MRPP (multi-response permutation procedure) based on Bray-Curtis dissimilarity distance was performed to evaluate the significance of orthologous profiles of microbial species against other species in the same genus. The vegan package [62] developed in R environment was used in this study.

## Abbreviations

ANI, average nucleotide identity; AAI, average amino acids identity; DDH, DNA-DNA hybridization; MLSA, multi-locus sequence analysis; MRPP, multi-response permutation procedure; PCoA, principle coordinate analysis; POCP, percentage of conserved proteins

## Additional files

Additional file 1: This file contains the supplementary table and figures for this paper, including Table S1, Figure S1, and Figure S2. (DOCX 262 kb) Additional file 2: This file contains the list of strain names and NCBI accession numbers for the microorganisms retrieved in this study. (XLSX 114 kb) Additional file 3: This file contains the perl script for generating orthologous gene profiles from usearch outputs against eggNOG (COG and NOG) database. (DOCX 15 kb) Additional file 4: This file contains the perl script for calculating pairwise Bray-Curtis dissimilarity among different microbial strains. (DOCX 15 kb)
