# Supplementary material for: Gene content dissimilarity for subclassification of highly similar microbial strains
Source: BMC Genomics. 2016 Aug 17;17:647. doi: 10.1186/s12864-016-2991-9 (PMC4988056; doi:10.1186/s12864-016-2991-9)
Supplement: Additional file 1: — This file contains the supplementary table and figures for this paper, including Table S1, Figure S1, and Figure S2. (DOCX 262 kb) [file 12864_2016_2991_MOESM1_ESM.docx]

Table S1. Significance tests of the orthologous gene profiles of microbial species against other species in the same genus. The non-parametric multivariate analysis MRPP (multi-response permutation procedure) based on Bray-Curtis dissimilarity distance was performed. Microbial strains in the genera with ≥2 species and each species with ≥5 strains were selected for significance tests.

| Genus | Species | δ^*^ | P |
| --- | --- | --- | --- |
| *Bacillus* | *Bacillus thuringiensis* | 0.234 | 0.001 |
|  | *Bacillus subtilis* | 0.223 | 0.001 |
|  | *Bacillus anthracis* | 0.25 | 0.002 |
|  | *Bacillus amyloliquefaciens* | 0.194 | 0.001 |
|  | *Bacillus cereus* | 0.233 | 0.001 |
| *Bifidobacterium* | *Bifidobacterium longum* | 0.097 | 0.001 |
|  | *Bifidobacterium animalis* | 0.097 | 0.001 |
| *Chlamydia* | *Chlamydia trachomatis* | 0.046 | 0.001 |
|  | *Chlamydia psittaci* | 0.046 | 0.001 |
| *Corynebacterium* | *Corynebacterium glutamicum* | 0.117 | 0.001 |
|  | *Corynebacterium pseudotuberculosis* | 0.125 | 0.001 |
|  | *Corynebacterium diphtheriae* | 0.131 | 0.001 |
| *Lactobacillus* | *Lactobacillus rhamnosus* | 0.275 | 0.003 |
|  | *Lactobacillus reuteri* | 0.224 | 0.001 |
|  | *Lactobacillus plantarum* | 0.237 | 0.001 |
|  | *Lactobacillus casei* | 0.255 | 0.001 |
| *Mycoplasma* | *Mycoplasma gallisepticum* | 0.154 | 0.001 |
|  | *Mycoplasma hyopneumoniae* | 0.182 | 0.001 |
|  | *Mycoplasma genitalium* | 0.199 | 0.001 |
|  | *Mycoplasma hyorhinis* | 0.191 | 0.001 |
| *Mycobacterium* | *Mycobacterium tuberculosis* | 0.097 | 0.002 |
|  | *Mycobacterium canettii* | 0.097 | 0.002 |
|  | *Mycobacterium bovis* | 0.096 | **0.024** |
| *Pseudomonas* | *Pseudomonas aeruginosa* | 0.221 | 0.001 |
|  | *Pseudomonas fluorescens* | 0.281 | **0.018** |
|  | *Pseudomonas stutzeri* | 0.24 | 0.001 |
|  | *Pseudomonas putida* | 0.243 | 0.001 |
| *Streptococcus* | *Streptococcus dysgalactiae* | 0.233 | 0.001 |
|  | *Streptococcus suis* | 0.207 | 0.001 |
|  | *Streptococcus pyogenes* | 0.197 | 0.001 |
|  | *Streptococcus thermophilus* | 0.223 | 0.001 |
|  | *Streptococcus pneumoniae* | 0.19 | 0.001 |
|  | *Streptococcus agalactiae* | 0.221 | 0.001 |

*Statistic δ is the overall weighted mean of within-group means of the pairwise dissimilarities among sampling units. The significance test is the fraction of permuted δ that is less than the observed δ.


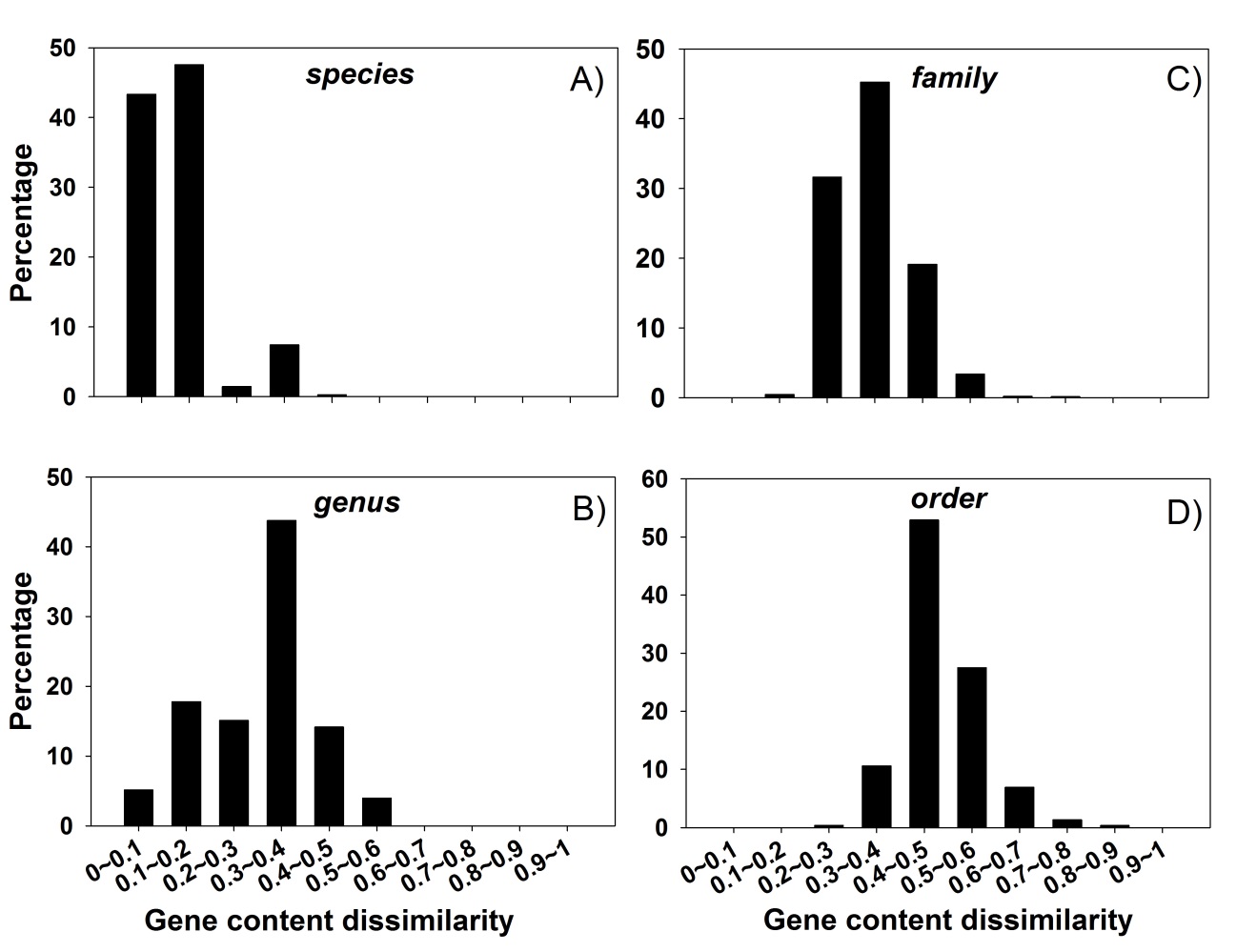


Figure S1. Distribution of gene content dissimilarity for the retrieved microbial genomes at different taxonomic levels, including species (A), genus (B), family (C), and order (D). Cutoffs of 0.2 and 0.4 were recommended for microbial species and family delineation, respectively. The eggNOG v4.5 database was used for orthologous group profiling.


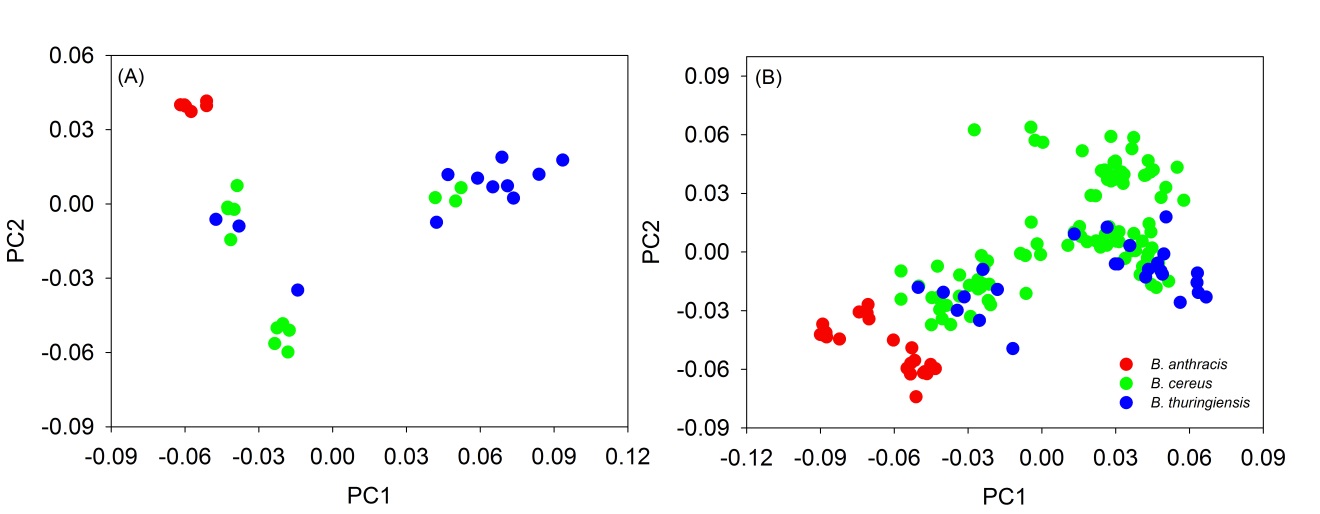


Figure S2. Application of gene content dissimilarity in classifying *Bacillus cereus* group strains. Two analyses were carried out here, including strains with complete genomes (A) and all strains with complete and draft genomes (B). Clear separation of *B. anthracis* from *B. cereus* and *B. thuringiensis* could be observed in both analyses.
