# Supplementary material for: Gene content dissimilarity for subclassification of highly similar microbial strains
Source: BMC Genomics. 2016 Aug 17;17:647. doi: 10.1186/s12864-016-2991-9 (PMC4988056; doi:10.1186/s12864-016-2991-9)
Supplement: Additional file 3: — This file contains the perl script for generating orthologous gene profiles from usearch outputs against eggNOG (COG and NOG) database. (DOCX 15 kb) [file 12864_2016_2991_MOESM3_ESM.docx]

#!/usr/bin/perl

use strict;

use Bio::SeqIO;

#NCBI taxonomic files (ftp://ftp.ncbi.nih.gov/pub/taxonomy/taxdump.tar.gz) and eggNOG member file (http://eggnogdb.embl.de/download/eggnog_4.5/data/NOG/NOG.members.tsv.gz) were required

#database searching was carried out againt the eggNOG COG and NOG members

my $nogmemfile = "NOG.members.tsv";

my $bacaafile = "bacteria_genome.faa"; #aa sequence file for all retrieved bacteria

my $usearchfile = "bacteria_genome.usearch"; #usearch results with blast output format

my $funprofile = "fun_profile.txt";

my %nogmap;

open( FILE, "$nogmemfile" ) || die "#1\n";

while (<FILE>) {

chomp;

my @items = split( "\t", $_ );

my @tmp = split( ",", $items[5] );

foreach my $id (@tmp) {

push( @{ $nogmap{$id} }, $items[1] );

}

}

close FILE;

my ( %species, %cog, %fun );

foreach my $fafile ("$bacaafile") {

my $seqIO = Bio::SeqIO->new( -file => "$fafile", -format => "fasta" );

while ( my $seqobj = $seqIO->next_seq ) {

my $id = $seqobj->id;

$id =~ /(.*?)\./;

my $species = $1;

if ( length( $seqobj->seq ) > 2 ) {

$species{$species} = 1;

if ( $nogmap{$id} ) {

my @cog = @{ $nogmap{$id} };

foreach my $cog (@cog) {

$fun{$species}{$cog}{$id} = 1;

$cog{$cog} = 1;

}

}

}

}

}

foreach my $ucfile ("$usearchfile") {

open( USEARCH, "$ucfile" ) || die "#3\n";

while (<USEARCH>) {

chomp;

my @items = split( "\t", $_ );

$items[0] =~ /(\d+)(\.|\_)/;

my $species = $1;

$species{$species} = 1;

if ( $nogmap{ $items[1] } ) {

my @cog = @{ $nogmap{ $items[1] } };

foreach my $cog (@cog) {

$fun{$species}{$cog}{ $items[0] } = 1;

$cog{$cog} = 1;

}

}

}

close USEARCH;

}

my @species = keys %species;

my @cogs = keys %cog;

my %lineage = &GetLineage(@species);

my @taxons = @species;

open( OUT, ">$funprofile" ) || die "#4\n";

print OUT "OGs\t", join( "\t", @taxons ), "\n";

foreach my $cog (@cogs) {

print OUT "$cog";

foreach my $species (@taxons) {

my $num = scalar( keys %{ $fun{$species}{$cog} } );

print OUT "\t$num";

}

print OUT "\n";

}

close OUT;

sub GetLineage() {

my @taxons = @_;

my ( %parents, %levels );

open( NODES, "taxonomy/nodes.dmp" ) || die "#1\n";

while (<NODES>) {

chomp;

my @items = split( "\t", $_ );

$parents{ $items[0] } = $items[2];

$levels{ $items[0] } = $items[4];

}

close NODES;

my %names;

open( FILE, "taxonomy/names.dmp" ) || die "#2\n";

while (<FILE>) {

chomp;

my @items = split( "\t", $_ );

$names{ $items[0] } = $items[2] if $items[6] eq "scientific name";

}

close FILE;

my %lineage;

foreach my $taxon (@taxons) {

my $child = $taxon;

$child =~ s/\s//g;

my $parent = $parents{$child};

while ( $child ne $parent ) {

$lineage{$taxon}{ $levels{$parent} } = $names{$parent}

if $levels{$parent} =~

/^(superkingdom|kingdom|phylum|class|order|family|genus|species)/;

$child = $parent;

$parent = $parents{$child};

}

}

return %lineage;

}
