# Supplementary material for: Gene content dissimilarity for subclassification of highly similar microbial strains
Source: BMC Genomics. 2016 Aug 17;17:647. doi: 10.1186/s12864-016-2991-9 (PMC4988056; doi:10.1186/s12864-016-2991-9)
Supplement: Additional file 4: — This file contains the perl script for calculating pairwise Bray-Curtis dissimilarity among different microbial strains. (DOCX 15 kb) [file 12864_2016_2991_MOESM4_ESM.docx]

**#!/usr/bin/perl**

**use strict;**

**use List::Util qw(sum);**

**my $funprofile="fun_profile.txt";**

**my $brayout="bray.txt";**

**my $sorenout="soren.txt";**

**my %cog;**

**my %sum;**

**open( FILE, "$funprofile" ) || die "#1\n";**

**my $line=<FILE>;**

**chomp $line;**

**my @heads=split("\t",$line);**

**while (<FILE>) {**

**chomp;**

**if ( $_ !~ /^#/ ) {**

**my @items = split( "\t", $_ );**

**for(my $i=1;$i<=$#items;$i++){**

**$cog{$heads[$i]}{$items[0]}+=$items[$i] if $items[$i]>0;**

**$sum{$heads[$i]}+=$items[$i];**

**}**

**}**

**}**

**close FILE;**

**#here only strains with >1000 mapped genes were analyzed**

**my @taxons;**

**foreach my $taxon(keys %sum){**

**push(@taxons,$taxon) if $sum{$taxon}>1000;**

**}**

**open(BRAY,">$brayout")||die"#2\n";**

**open(SOREN,">$sorenout")||die"#3\n";**

**my %dissimilarity;**

**print BRAY "\t",join("\t",@taxons),"\n";**

**print SOREN "\t",join("\t",@taxons),"\n";**

**foreach my $species1 ( @taxons ) {**

**print BRAY "$species1";**

**print SOREN "$species1";**

**foreach my $species2 ( @taxons ) {**

**if (!$dissimilarity{$species1}{$species2})**

**{**

**print "$species1\t$species2\n";**

**$dissimilarity{$species1}{$species2} = 1;**

**my $bray = &CalBray( \%{ $cog{$species1} }, \%{ $cog{$species2} } );**

**my $soren = &CalSoren( \%{ $cog{$species1} }, \%{ $cog{$species2} } );**

**print BRAY "\t$bray";**

**print SOREN "\t$soren";**

**}**

**}**

**print BRAY "\n";**

**print SOREN "\n";**

**}**

**close SOREN;**

**close BRAY;**

**sub CalBray() {**

**my ( $hash1, $hash2 ) = @_;**

**my %hash1 = %$hash1;**

**my %hash2 = %$hash2;**

**my $common;**

**foreach my $key ( keys %hash1 ) {**

**if ( $hash2{$key} ) {**

**my $value = ( $hash1{$key} <= $hash2{$key} ) ? $hash1{$key} : $hash2{$key};**

**$common += $value;**

**}**

**}**

**my $hash1size;**

**foreach my $key ( keys %hash1 ) {**

**$hash1size += $hash1{$key};**

**}**

**my $hash2size;**

**foreach my $key ( keys %hash2 ) {**

**$hash2size += $hash2{$key};**

**}**

**my $dissimilarity = 1 - 2 * $common / ( $hash1size + $hash2size );**

**return $dissimilarity;**

**}**

**sub CalSoren() {**

**my ( $hash1, $hash2 ) = @_;**

**my %hash1 = %$hash1;**

**my %hash2 = %$hash2;**

**my $common;**

**foreach my $key ( keys %hash1 ) {**

**$common++ if $hash2{$key};**

**}**

**my $hash1size = scalar( keys %hash1 );**

**my $hash2size = scalar( keys %hash2 );**

**my $dissimilarity = 1 - 2 * $common / ( $hash1size + $hash2size );**

**return $dissimilarity;**

**}**
